# Th1 Cytokine-Secreting Recombinant *Mycobacterium bovis* Bacillus Calmette-Guérin and Prospective Use in Immunotherapy of Bladder Cancer

**DOI:** 10.1155/2011/728930

**Published:** 2011-09-15

**Authors:** Yi Luo, Jonathan Henning, Michael A. O'Donnell

**Affiliations:** Department of Urology, University of Iowa, 375 Newton Road, 3202 MERF, Iowa City, IA 52242, USA

## Abstract

Intravesical instillation of *Mycobacterium bovis* bacillus Calmette-Guérin (BCG) has been used for treating bladder cancer for 3 decades. However, BCG therapy is ineffective in approximately 30–40% of cases. Since evidence supports the T helper type 1 (Th1) response to be essential in BCG-induced tumor destruction, studies have focused on enhancing BCG induction of Th1 immune responses. Although BCG in combination with Th1 cytokines (e.g., interferon-**α**) has demonstrated improved efficacy, combination therapy requires multiple applications and a large quantity of cytokines. On the other hand, genetic manipulation of BCG to secrete Th1 cytokines continues to be pursued with considerable interest. To date, a number of recombinant BCG (rBCG) strains capable of secreting functional Th1 cytokines have been developed and demonstrated to be superior to BCG. This paper discusses current rBCG research, concerns, and future directions with an intention to inspire the development of this very promising immunotherapeutic modality for bladder cancer.

## 1. Clinical Use of BCG in Bladder Cancer Treatment

Urothelial carcinoma of the bladder is the second most common urologic neoplasm after prostate carcinoma in the United States, with an estimated 70,530 new cases and 14,680 deaths in 2010 [1]. Global prevalence of bladder cancer is estimated at >1 million and is steadily increasing. At the time of diagnosis, 20–25% of cases are muscle invasive (stage T2 or higher) and are typically treated with surgical resection (radical cystectomy) [2]. The remainders are nonmuscle invasive bladder cancer (NMIBC) including tumors confined to the epithelial mucosa (Ta), tumors invading the lamina propria (T1), and carcinoma *in situ *(Tis). Transurethral resection of bladder tumor (TURBT) is the primary treatment for Ta and T1 lesions. Intravesical therapy is used as adjuvant treatment to prevent recurrence and progression of the disese after TURBT and is also the treatment of choice for carcinoma *in situ.* Intravesical administration of BCG, a live attenuated strain of *Mycobacterium bovis* widely used as a vaccine against tuberculosis, is currently the most common therapy employed for NMIBC. Since its advent in 1976 [3], BCG has been extensively used to reduce recurrence and progression of NMIBC in an attempt to preserve the bladder. BCG therapy results in 50–60% effectiveness against small residual tumors and a 70–75% complete response rate for carcinoma *in situ*. Adjuvant intravesical therapy was noted by the 2007 American Urological Association (AUA) panel to reduce recurrences by 24% and treatment with BCG was recommended by the panel. Unfortunately, a high percentage of patients fail initial BCG therapy and 40–50% of BCG responders develop recurrent tumors within the first 5 years [2]. In addition, up to 90% of patients experience some sort of side effects including, although rare, life-threatening complications such as sepsis.

According to the AUA's 2007 clinical practice guidelines, BCG therapy should be initiated two to three weeks following TURBT with a classic course consisting of six weekly intravesical installations. Lyophilized powder BCG (81 mg corresponding to 1–5 × 10^8^ colony-forming units of viable mycobacteria) is reconstituted in 50 mL of saline and administered via urethral catheter into an empty bladder with a dwell time of 2 hours. Maintenance BCG is more effective in decreasing recurrence as compared to induction therapy alone. Multiple meta-analyses support BCG maintenance and it is now firmly established in clinical practice. The European Association of Urology (EAU) and the AUA recommend one year of maintenance for high-risk patients [4, 5]. An optimal schedule/duration of therapy has yet to be determined; however, most who use maintenance follow some permutation of the Southwest Oncology Group (SWOG) program, a 3-week “mini” series given at intervals of 3, 6, 12, 18, 24, 30, and 36 months [6]. At our own institution, induction (first BCG therapy) is initiated 2 to 3 weeks following TURBT with 6 weekly installations and a 1-2 hour dwell time. For patients with carcinoma *in situ*, severe dysplasia, Grade 3/high grade or poorly differentiated pathology, and/or stage T1 disease, formal restaging under anesthesia is performed 6 weeks later including obtaining bilateral upper tract cytology, retrograde pyelograms, 4-5 random bladder biopsies, and prostatic urethral biopsies. If this pathology and restaging is negative, maintenance cycles may be initiated in 6 weeks. We classify three maintenance cycles A, B, and C. Maintenance A consists of 3 weekly instillations followed by cystoscopy 6 weeks later. Cytology and fluorescence *in situ* hybridization (FISH) in urine specimens may be obtained at this time. If cystoscopy/cytology is negative, maintenance B may be initiated 6 months after the conclusion of cycle A, again for 3 weekly treatments. Maintenance C is initiated 6 months after the conclusion of cycle B. Following cycle C, cystoscopy/cytology is repeated every 3 months for 2 years from the original diagnosis at which time it is extended to every 6 months for 1 year, and then annually.

## 2. Mechanism of BCG Action

Since its first therapeutic application in 1976, major efforts have been made to decipher the mechanisms through which BCG mediates antibladder cancer immunity [7, 8]. During the past decades, many details of the molecular and cellular mechanisms involved have been discovered although the exact mechanisms of BCG action still remain elusive. It is now accepted that a functional host immune system is a necessary prerequisite for successful BCG immunotherapy. It has also become clear that the effects of intravesical BCG depend on the induction of a complex inflammatory cascade event in the bladder mucosa reflecting activation of multiple types of immune cells and bladder tissue cells [7, 8]. After instillation, BCG adheres to fibronectin on the urothelial lining through a fibronectin attachment protein (FAP) on BCG [9]. This interaction between BCG and the urothelium is one of the first and most crucial steps. Attached BCG is then internalized and processed by urothelial cells including urothelial carcinoma cells (UCCs), resulting in secretion of an array of proinflammatory cytokines and chemokines such as interleukin (IL)-1, IL-6, IL-8, tumor necrosis factor (TNF)-*α*, and granulocyte-macrophage colony stimulating factor (GM-CSF) [10, 11]. Following urothelial cell activation, an influx of various leukocyte types into the bladder wall occurs including neutrophils, monocytes/macrophages, lymphocytes, natural killer (NK) cells, and dendritic cells (DCs) [12–14]. These infiltrating leukocytes are activated and produce a variety of additional proinflammatory cytokines and chemokines and also form BCG-induced granuloma structures in the bladder wall [12, 14]. Subsequently, a large number of leukocyte types such as neutrophils, T cells, and macrophages are expelled into the bladder lumen and appear in patients' voided urine [15–18]. In addition, transient massive cytokines and chemokines can be detected in voided urine including IL-1*β*, IL-2, IL-6, IL-10, IL-12, IL-18, interferon (IFN)-*γ*, TNF-*α*, GM-CSF, macrophage colony-stimulating factor (M-CSF), macrophage-derived chemokine (MDC), monocyte chemoattractant protein (MCP)-1, macrophage-inflammatory-protein- (MIP-) 1*α*, interferon-inducible protein (IP)-10, monokine induced by *γ*-interferon (MIG), and eosinophil chemoattractant activity (Eotaxin) [17, 19–24]. The urine of animals treated with intravesical BCG also showed increased IL-1*α*, IL-1*β*, IL-2, IL-3, IL-4, IL-5, IL-6, IL-10, IL-12, IL-17, IFN-*γ*, TNF-*α*, GM-CSF, M-CSF, and MIP-1*α*, regulated on activation normal T cell expressed and secreted (RANTES), and keratinocyte-derived chemokine (KC) [14]. It has been noted that the development of a predominant Th1 cytokine profile (e.g., IFN-*γ*, IL-2, and IL-12) is associated with the therapeutic effects of BCG, whereas the presence of a high level of Th2 cytokines (e.g., IL-10) is associated with BCG failure [20, 22, 23]. Thus, a shift of the cytokines produced towards a Th1 milieu is necessary for succesful BCG immunotherapy of bladder cancer. To support this, it has been observed that both IFN-*γ* and IL-12 but not IL-10 are required for local tumor surveillance in an animal model of bladder cancer [25]. Mice deficient in IL-10 genetically (IL-10^−/−^) or functionally via antibody neutralization can also develop enhanced antibladder cancer immunity in response to intravesical BCG [23].

Multiple immune cell types participate in the inflammatory response induced by BCG in the bladder. It is well accepted that macrophages, an indispensable cellular component of the innate immune system, serve as the first line of defense in mycobacterial infection. Activation, maturation, and cytokine production of macrophages are primarily induced by Toll-like receptor (TLR) 2 ligation [26]. Following BCG instillation, an increased number of macrophages can be observed in bladder cancer infiltrates and the peritumoral bladder wall. Voided urine after BCG instillation also contains an increased number of macrophages and the cytokines and chemokines predominantly produced by macrophages such as TNF-*α*, IL-6, IL-10, IL-12, and IL-18 [15, 17, 19, 22–24]. In addition to presenting BCG antigens, both human and murine macrophages are capable of functioning as tumoricidal cells toward bladder cancer cells upon activation by BCG *in vitro* [27–31]. The killing of bladder cancer cells by macrophages relies on direct cell-to-cell contact and release of various soluble effector factors such as cytotoxic cytokines TNF-*α* and IFN-*γ* and apoptotic mediators such as nitric oxide (NO) [29–32]. Th1 cytokines (e.g., IFN-*γ*) enhance the induction of macrophage cytotoxicity whereas Th2 cytokines (e.g., IL-10) inhibit the induction of macrophage cytotoxicity [30, 31]. 

Neutrophils also compose the early responding cells to BCG instillation of the bladder and can be observed in the bladder wall and urine shortly after BCG instillation [14, 15, 17, 18]. Neutrophils are central mediators of the innate immunity in BCG infection and are activated by signalling through TLR2 and TLR4 in conjunction with the adaptor protein myeloid differentiation factor 88 (MyD88) [33]. In addition to secretion of proinflammatory cytokines and chemokines (e.g., IL-1*α*, IL-1*β*, IL-8, MIP-1*α*, MIP-1*β*, MCP-1, transforming growth factor (TGF)-*β*, and growth-related oncogene (GRO)-*α*) that lead to the recruitment of other immune cells [34], recent studies revealed that neutrophils are the primary source of TNF-related apoptosis-inducing ligand (TRAIL) found in the urine after BCG instillation [35, 36]. TRAIL is a member of the TNF family that induces apoptosis in malignant cells but not in normal cells. Studies have indicated that the neutrophil TRAIL response is specific to BCG stimulation rather than nonspecific immune activation. Studies have also revealed a positive correlation between urinary TRAIL level and the therapeutic effects of BCG, as BCG responders contained a significant higher amount of urinary TRAIL than BCG nonresponders [35]. These observations suggest an important role of neutrophils in BCG-induced antibladder cancer immunity. Indeed, it has been observed that depletion of neutrophils resulted in a reduced BCG-induced antibladder cancer response in a mouse model of bladder cancer [34]. 

Following the activation of macrophages and neutrophils in the bladder wall, driven by chemoattractants, recruitment of other immune cell types including CD4^+^ T cells, CD8^+^ T cells, NK cells, and DC takes place [12, 13]. As for neutrophils and macrophages, these cell types can be found in the voided urine of patients after BCG instillation [15–17]. These effector cells produce various cytokines and chemokines to further promote BCG-induced antibladder cancer immune responses in the local milieu. In addition, DC, together with macrophages, trigger an anti-BCG-specific immune response via antigen presentation to T cells that also amplifies the BCG-induced antitumor immunity. Like neutrophils and macrophages, both T cells and NK cells are cytotoxic toward bladder cancer cells upon activation. They kill target cells via the major histocompatibility complex (MHC) restricted (e.g., for cytotoxic T lymphocytes (CTL)) and/or MHC nonrestricted pathways (e.g., for NK cells) [27, 37, 38]. Perforin-mediated lysis and apoptosis-associated killing (e.g., via Fas ligand and TRAIL) have been implicated as the major molecular effector mechanisms underlying the eradication of bladder cancer cells. These effector cell types are crucial for BCG immunotherapy of bladder cancer, as depletion of these cell types failed to develop effective antibladder cancer responses *in vivo* and kill bladder cancer cells *in vitro* [39, 40].

It has been shown that stimulation of human peripheral blood mononuclear cells (PBMCs) by viable BCG *in vitro* leads to the generation of a specialized cell population called BCG-activated killer (BAK) cells [41, 42]. BAK cells are a CD3^−^CD8^+^CD56^+^ cell population whose cytotoxicity is MHC nonrestricted [42, 43]. BAK cells kill bladder cancer cells through the perforin-mediated lysis pathway and effectively lyse NK cell-resistant bladder cancer cells [41–43]. Macrophages and CD4^+^ T cells have been found to be indispensable for the induction of BAK cell killing activity but have no such activity by themselves [42]. Th1 cytokines IFN-*γ* and IL-2 have also been found to be required for the induction of BAK cell cytotoxicity, as neutralizing antibodies specific to these cytokines could inhibit BCG-induced cytotoxicity [42]. BAK cells, together with lymphokine-activated killer (LAK) cells, a diverse population with NK or T-cell phenotypes that are generated by IL-2 [44, 45], have been suggested to be the major effector cells during intravesical BCG immunotherapy of bladder cancer. Other potential cytotoxic effector cells include CD1 restricted CD8^+^ T cells [46], *γδ* T cells [47], and natural killer T (NKT) cells [47, 48].

Activation of the innate immune system is a prerequisite for the BCG-induced inflammatory responses and the subsequent eradication of bladder cancer by intravesical BCG. In BCG instillation, TLRs participate in neutrophil, macrophage and DC maturation and activation. Both TLR2 and TLR4 appear to serve important but distinct roles in the induction of host immune responses to BCG or BCG cell-wall skeleton [26]. Like other microbes, BCG has surface components called pathogen-associated molecular patterns (PAMPs) that are recognized by cells of the innate immune system through TLRs during infection [49]. It is this interaction between TLRs and PAMPs that activates the cells of the innate immune system, leading to BCG-induced inflammatory responses and subsequent eradication of bladder cancer. It is known that the antitumor effect of intravesical BCG depends on its proper induction of a localized Th1 immune response. However, a systemic immune response also appears involved in intravesical BCG therapy. It has been reported that purified protein derivative (PPD) skin test often converts from negative to positive after BCG instillation and the effective treatment is associated with the development of delayed-type hypersensitivity (DTH) reaction to PPD [50]. Animal studies have also demonstrated the importance of DTH in the antitumor activity of intravesical BCG therapy [23]. Moreover, studies have shown increased levels of cytokines and chemokines in the serum (e.g., IL-2, IFN-*γ*, MCP-1, and RANTES), along with production of these cytokines and chemokines in the urine and/or bladder, during the course of BCG instillation [21, 51]. Furthermore, studies have also shown an increase in PBMC cytotoxicity against UCC after BCG instillation [21]. 

In addition to the ability of BCG to elicit host immune responses, evidence supports a direct effect of BCG on the biology of UCC. *In vitro* studies have shown that BCG is antiproliferative and even cytotoxic to UCC [27, 52] and induces UCC expression of cytokines and chemokines (e.g., IL-1*β*, IL-6, IL-8, TNF-*α*, and GM-CSF) [11], antigen-presenting molecules (e.g., MHC class II, CD1 and B7-1) [53], and intercellular adhesion molecules (e.g., ICAM-1) [53]. Analysis of tumor biopsy specimens from bladder cancer patients who underwent intravesical BCG therapy further supported the ability of BCG to induce UCC expression of these molecules *in vivo *[13]. Moreover, the bladder urothelium of animals treated with intravesical BCG shows upregulation of HLA antigens (e.g., MHC class I and II) and changes of many other molecules [54]. Recent studies have revealed that by cross-linking *α*5*β*1 integrin receptors, BCG exerts its direct biological effects on UCC, including activation of the signal transduction pathways involving activator protein (AP) 1, NF*κ*B and CCAAT-enhancer-binding protein (C/EBP) [55], upregulation of gene expressions such as IL-6 and cyclin dependant kinase inhibitor p21 [55, 56], and cell cycle arrest at the G1/S transition [57]. Although some studies showed the ability of BCG to induce apoptosis in UCC [58], other studies demonstrated that BCG induced no apoptosis or even caused apoptotic resistance in UCC [59]. Further studies revealed that BCG induced UCC death in a caspase-independent manner [59] and that p21 played an important role in modulating the direct effects of BCG on UCC [60].

## 3. Combination of BCG with Th1 Cytokines for Bladder Cancer Treatment

The proper induction of Th1 immunity is required for successful BCG immunotherapy of bladder cancer. Since a high percentage of patients do not respond to BCG and the effect of BCG is associated with significant toxicity, strategies to combine BCG with recombinant (*r*) Th1 cytokines to enhance BCG therapeutic efficacy while reducing BCG toxicity have been employed and studied. Among Th1 cytokines, rIFN-*α* is most extensively studied and has been shown to be safe and tolerable when used intravesically, alone or in combination with BCG, in many controlled studies [61–65]. The side-effect profile of combination therapy is similar to BCG monotherapy including lower urinary tract symptoms such as frequency, urgency, dysuria, bladder spasm, and hematuria. Systemic fever, flu-like symptoms, and myalgias were found in <25% of patients and were self-limited. Benefits have been seen in patients with BCG failures [61–63]. Treatment with low-dose BCG (1/3 or 1/10 the standard dose) combined with rIFN-*α* resulted in 45–53% of patients who had failed prior BCG monotherapy to remain disease-free at 24-month median followup [61, 63]. The benefit in naïve patients is currently in question with recent studies showing mixed results. A Phase III study suggested no benefit in BCG naïve patients [64]. However, no subgroup analysis was performed for carcinoma *in situ* or high-risk patients. Therefore, it can still be concluded that the BCG-rIFN-*α* combination therapy may provide a benefit to patients with high-risk disease or carcinoma *in situ*. Data since the release of the Phase III study supports the combination therapy with BCG and rIFN-*α* in BCG naïve patients [65]. Thus, more studies are needed to formally determine the effect of the combination therapy for BCG naive patients. To date, a combination therapy with BCG and rIFN-*α*2B has been employed, particularly for patients with previous BCG failures, those with carcinoma *in situ*, and the elderly [63]. Optimal dose and schedule have yet to be defined in controlled trials and debate continues on the subject. At our institution, we use the standard dose of TICE BCG plus 50 million units (MU) of rIFN-*α*2B intravesically as induction therapy for BCG naïve patients. For BCG exposed patients, 1/3 the standard dose of BCG plus 50 MU of rIFN-*α*2B is utilized. The dose may be lowered for those patients experiencing lower urinary tract symptoms or low grade fever. For maintenance cycle A, we adjust the BCG dose for week 1 consisting of 1/3 the standard dose of BCG plus 50 MU of rIFN-*α*2B. For weeks 2 and 3, the BCG dose is lowered to 1/10 the standard dose plus 50 MU of rIFN-*α*2B. Maintenance cycles B and C utilize similar dosing.

Other cytokines that have been used intravesically include rIL-2, rIL-12, rIFN-*γ*, and rGM-CSF. A study demonstrated that intravesical rIL-2 was beneficial for patients with T1 papillary bladder carcinoma after TURBT showing regression of marker lesions and lack of major toxic effects [66]. Other studies also demonstrated intravesical rIL-2 to be feasible, safe, and effective in patients with NMIBC who were untreated or had failed prior intravesical therapy with other agents [67, 68]. A study demonstrated that intravesical rIL-12 was well tolerated by patients with recurrent NMIBC but showed no clinically relevant antitumor and immunologic effects [69]. However, the maximum tolerated dose of rIL-12 was not reached in the study. Different from human studies, animal studies showed encouraging results. A survival advantage of intravesical rIL-12 was observed in a mouse orthotopic bladder cancer model [70]. Further studies for intravesical rIL-12 use are warranted. For intravesical rIFN-*γ*, a study showed the absence of major toxicity and the therapeutic effect superior to mitomycin C for patients with NMIBC who underwent TURBT [71]. In addition, populations of leukocytes in the urothelium were significantly increased in rIFN-*γ*-treated patients confirming its induction of localized cellular immune responses. Other studies also supported the safety and antitumor activity of intravesical rIFN-*γ* monotherapy [72]. Studies also demonstrated that intravesical rGM-CSF was effective as a prophylactic therapy for patients with NMIBC after TURBT [73, 74]. In correlation with regression of marker lesions, intravesical rGM-CSF induced leukocyte migration and activation in the bladder mucosa. Despite all these observations, however, single cytokine therapy has only been evaluated in small numbers of patients and has not yet shown compelling results in general. Indeed, *in vitro* studies have demonstrated that cytokines IL-2, IL-12, and TNF-*α*, like IFN-*α*, can enhance BCG for the induction of Th1 immune responses in human PBMC [75–77]. Thus, addition of these cytokines to BCG may provide benefits for BCG therapy particularly for BCG nonresponders or relapsers. Studies are absolutely needed to examine the combination of BCG with these cytokines for the treatment of bladder cancer.

## 4. Advances in Genetic Engineering of BCG for Cytokine Delivery

### 4.1. BCG as a Heterologous Gene Delivery Vehicle

Because of its unique characteristics, such as adjuvant potential, low toxicity, and potent immunogenicity, BCG has long been considered to be an attractive live vaccine delivery vehicle with which to deliver protective antigens of multiple pathogens. During the past 2 decades, with advances in knowledge of mycobacterial genetics and molecular biology, a wide range of rBCG vaccine candidates expressing bacterial, viral, parasitic antigens have been developed including those for *Mycobacterium tuberculosis *(*M.tb*), human immunodeficiency virus (HIV), and hepatitis B and C viruses [78]. As early as in the 1980s, studies showed that mycobacteria were capable of delivering foreign genes that were introduced into the microbes [79, 80]. In the early 1990s, vectors carrying strong promoters from the mycobacterial major heat-shock protein genes (e.g., *hsp60* and *hsp70*) and unique cloning sites, which allowed extrachromosomal or integrative expression of foreign antigens, were developed [81, 82]. Using these expression vectors, BCG was further demonstrated to be an effective live delivery vehicle for foreign antigens [81, 83–87]. These rBCG strains constitutively expressed foreign antigens and elicited long-lasting specific humoral and/or cellular immune responses in mice. Some of these rBCG strains even generated protective immunity against respective pathogens whose antigens were expressed by mycobacteria such as the outer surface protein A (OspA, *Borrelia burgdorferi*) [83], surface proteinase gp63 (*Leishmania spp*) [85], and surface protein A (*Streptococcus pneumoniae*) [86]. During that time period, vectors permitting surface expression of foreign antigens in mycobacteria or secretion from mycobacteria were also developed [83, 88]. Infection with these rBCG strains led to enhanced immune responses to some antigens in mice [83, 86, 89]. Meanwhile, vectors with various mycobacterial gene promoters, such as *α*-antigen, P_AN_, ag85b, 18 kDa, and furA (among many others), were also developed and demonstrated to be effective to elicit specific immune responses and/or protective immunity in different animal species including mouse, guinea pig, hamster, pig, sheep, rabbit, and monkey [78, 88, 90–92]. In addition, progress has continued in the refinement of the safety and efficacy of the rBCG vaccine vehicles. To date, numerous improved systems employed to express heterologous genes in BCG are available. Among them are vectors with limited replication or auxotrophic complementation for safe use in HIV-infected individuals, capability to replicate at a high-copy number for increased antigen delivery, dual expression cassettes for multivalent antigen delivery, capability to integrate into the genome at multiple sites for differential antigen expression, inducible elements for controlled gene expression, and expression of perfringolysin or listeriolysin (with or without urease C gene deletion) for increased CD8^+^ T-cell stimulation. Although clinical use of rBCG vaccines is still in an early stage, studies have already demonstrated that rBCG is safe and effective in humans such as those expressing OspA and *M.tb* antigen 85B (Ag85B). In the years to come, more rBCG vaccines will be evaluated clinically and their usefulness in preventing human infectious dieases will become clear. 

In addition to a wide range of bacterial, viral, and parasitic antigens, BCG has also been engineered to deliver tumor-associated antigens. For example, BCG expressing prostate specific molecules such as prostate specific antigen (PSA) and prostate specific membrane antigen (PSMA) have been developed. Mice immunized with the rBCG-PSA or rBCG-PSMA strain developed antigen-specific immune responses, primarily a cellular immune response [93]. We also independently developed a rBCG strain that secretes the full-length PSA. We observed that mice immunized with the rBCG-PSA strain, but not a control BCG strain carrying an empty vector, developed a potent specific CTL activity against PSA-expressing RM11psa cells (our unpublished observations). In addition, we further observed that mice primed with the rBCG-PSA strain and boosted with Ad-PSA, a replication-defective adenoviral vector carrying the full-length PSA coding sequence [94], developed enhanced PSA-specific CTL activity and IFN-*γ* expressing CD4^+^ and CD8^+^ T cells (our unpublished observations). Several studies including ours have also demonstrated that BCG could be engineered to express mucin-1 (MUC1), a candidade tumor-associated antigen for breast cancer and other epithelial adenocarcinomas, in a manner of multiple tandem repeats with coexpression of IL-2, GM-CSF, or CD80 [95–99]. Severe combined immunodeficient (SCID) mice reconstituted with human peripheral blood lymphocytes (PBLs) followed by immunization with these MUC1-expressing rBCG strains developed specific protective immunity against MUC1-positive human breast cancer xenografts. These observations warrant further studies in rBCG delivering tumor antigens for the treatment of malignant diseases. 

Studies have shown that BCG delivery of certain biologically active molecules can induce enhanced immune responses. A study demonstrated that a rBCG strain secreting cathepsin S, a cysteine endoprotease involved in MHC class II antigen presentation, could restore intracellular cathepsin S activity and improve the capacity of BCG-infected macrophages to stimulate CD4^+^ T cells [121]. A study also demonstrated that mice simultaneously immunized with intraperitoneal ovalbumin (OVA) and intranasal rBCG secreting the assembled pentameric cholera toxin B subunit developed a long-lasting OVA-specific mucosal IgA response as well as a systemic IgG response [122]. Remarkably, a rBCG strain expressing the genetically detoxified S1 subunit of pertussis toxin (S1PT) showed enhanced BCG adjuvant potential and, when administered intravesically, resulted in bladder weight reduction and increased survival time in a mouse syngeneic orthotopic tumor model [123, 124]. Moreover, BCG has also been engineered to express the model antigen OVA for studies of the mechanisms underlying BCG induction of antigen-specific immune responses [125]. These studies revealed that the ability of BCG to induce a delayed but persistent immune response was due to its chronicity in infection that led to a long effector phase and reduced immune cell attrition compared to* Listeria monocytogenes* (an acute pathogen). Furthermore, we and others have also engineered BCG to express green fluorescent protein (GFP), either alone or in combination with antigenic molecules (e.g., OVA) or cytokines (e.g., IL-2), for the studies of BCG trafficking, antigen deliver, and antimycobacterial infection [109, 126, 127]. 

### 4.2. Th1 Cytokine-Secreting rBCG

In our early studies, we developed a panel of rBCG strains that secreted mouse IL-2 or rat IL-2 under the control of the mycobacterial *hsp60* promoter and *α*-antigen signal sequence [100]. We demonstrated that the IL-2 secreting rBCG strains induced enhanced IFN-*γ* production by mouse splenocytes *in vitro* compared to wild-type BCG. Since then, numerous rBCG strains secreting different mouse and human cytokines, primarily Th1 cytokines (e.g., IL-2, IL-18, IFN-*γ*, and IFN-*α*), have been developed (Table 1). In addition, rBCG strains secreting other cytokines or chemokines (e.g., GM-CSF, IL-15, TNF-*α*, and MCP-3) have also emerged. Most of these cytokine- and chemokine-secreting rBCG strains showed their abilities to enhance BCG-induced cellular immune responses including Th1 cytokine production, cellular cytotoxicity, DC activation, and anti-BCG or anti-*M.tb* infection. Some of them even showed their antitumor effects in animal models of melanoma [101], breast cancer [96, 97, 99], and bladder cancer [115]. Certain cytokine-secreting rBCG strains also induced humoral immune responses and Th2 cytokine production other than cellular immune responses *in vitro* and *in vivo*.

## 5. Th1 Cytokine-Secreting rBCG in Cancer Treatment

### 5.1. Antitumor Studies

BCG is a potent immunoadjuvant and induces a Th1 predominant immune response that is required for effective tumor eradication in most cancer types. Genetic manipulation of BCG to secrete Th1-stimulating cytokines with simultaneous coexpression of tumor-associated antigens may therefore potentiate the induction of specific antitumor immune responses. This strategy has been approached since the emergence of cytokine-secreting rBCG strains in the 1990s. Early studies demonstrated that mouse IL-2 secreting rBCG was at least equally effective to wild-type BCG when used as an intratumoral injection or a vaccine therapy in conjunction with irradiated tumor cells in a mouse melanoma model [101]. However, it was not until recently that the potential of rBCG for treating cancer has gained further appreciation. We and others have developed rBCG strains that deliver the breast-cancer-associated antigen MUC1 in a form of multiple tandem repeats with coexpression of human IL-2 or human GM-CSF [95–97, 99]. SCID mice reconstituted with human PBL followed by immunization with the rBCG strains developed MUC1-specific cellular immune respnses and enhanced protection against MUC1-positive human breast cancer xenografts compared to control mice reconstituted with human PBL and immunized with noncytokine secreting BCG. Studies have also demonstrated that the antitumor effects of the rBCG strains were correlated with the number of MUC1 tandem repeats delivered by BCG [97, 99]. These results suggest that these MUC1 rBCG strains coexpressing Th1-stimulating cytokines are promising candidates as breast cancer vaccines and thus deserve further investigation.

### 5.2. Antibladder Cancer Studies

Intravesical BCG is currently the treatment of choice for NMIBC. As for most other cancer types, the proper induction of a cellular immune response is required for successful BCG immunotherapy of bladder cancer. Studies have demonstrated that Th1 cytokine-secreting rBCG strains are capable of inducing enhanced cellular immune responses, leading to effective protection against mycobacterial infection (e.g., *M.tb*) and tumor progression (e.g., breast cancer) in various animal models. Unfortunately, studies on rBCG for treating bladder cancer are currently underdeveloped and, up to date, only a few reports have been available. However, studies have demonstrated that Th1 cytokine-secreting rBCG strains are superior to noncytokine secreting BCG for the induction of antibladder cancer immune responses *in vitro* and *in vivo*. 

#### 5.2.1. In Vitro Studies

It has been known that BCG stimulation of human PBMC leads to the generation of effector cells cytotoxic to bladder cancer cells *in vitro* [41, 42]. We recently demonstrated that stimulation of human PBMC with rBCG-IFN-*α*, a rBCG strain secreting human IFN-*α*2B [111], *in vitro* for 7 days induced enhanced PBMC cytotoxicity toward human bladder cancer cell lines T24, J82, 5637, TCCSUP, and UMUC-3 by up to 2-fold compared to control BCG carrying an empty vector [38]. This induction of enhanced PBMC cytotoxicity was correlated with increased production of IFN-*γ*, and IL-2 by rBCG-stimulated PBMC. Studies further revealed that this enhancement in PBMC cytotoxicity was dependent on BCG secreted IFN-*α* as well as endogenously expressed IFN-*γ* and IL-2, as blockage of IFN-*α*, IFN-*γ* or IL-2 by neutralizing antibodies during BCG stimulation reduced or abolished the induction of this enhanced PBMC cytotoxicity. Studies using NK and CD8^+^ T cells isolated from human PBMC revealed that both cell types were responsible for the enhanced PBMC cytotoxicity induced by rBCG-IFN-*α* with the former cell type being more predominant. 

An early study demonstrated that human peripheral monocytes/macrophages were capable of functioning as tumoricidal cells toward bladder cancer UCRU-BL-17 cells upon activation by BCG *in vitro* [27]. It was observed that the cytotoxic activity of human monocytes/macrophages was significantly enhanced after BCG stimulation, while the naïve cells exhibited only minimum cytotoxicity. Later, more studies including ours further demonstrated that mouse macrophages could also function as tumoricidal cells toward bladder cancer cells upon activation by BCG *in vitro* [28–31]. Stimulation of thioglycollate-elicited peritoneal macrophages by BCG for 24 hour resulted in macrophage-mediated killing of bladder cancer MBT-2 (C3H background) and MB49 (C57BL/6 background) cells in a dose-dependent manner [30, 31]. Studies also revealed that endogenous Th1 cytokines (e.g., IL-12, IL-18, IFN-*γ*, and TNF-*α*) played an important role in BCG-induced macrophage cytotoxicity, as blockage of these cytokines during BCG stimulation led to substantially reduced macrophage cytotoxicity toward bladder cancer cells [30]. In contrast, supplementation of BCG with Th1 cytokines (e.g., rIL-2, rIL-12, or rIL-18) increased macrophage cytotoxicity by approximately 2-fold. Consistent with these observations, rBCG strains secreting mouse IL-2 or mouse IL-18 showed enhanced macrophage-mediated killing on bladder cancer MBT-2 cells, which was correlated with increased expression of IFN-*γ*, TNF-*α*, and IL-6 by rBCG-stimulated macrophages [30]. The effect of mouse IL-2 secreting rBCG strain on the induction of macrophage cytotoxicity toward bladder cancer MBT-2 cells was also demonstrated by a separate study [28].

#### 5.2.2. In Vivo Studies

Although the *in vitro* studies have suggested the potential usefulness of Th1 cytokine-secreting rBCG strains for the treatment of bladder cancer, the effect of rBCG on treating bladder cancer *in vivo* has not well been studied. Up to date, only an rBCG strain secreting mouse IFN-*γ* (rBCG-IFN-*γ*) has been studied in a mouse MB49 syngeneic orthotopic tumor model [115]. This study showed that, with a low-dose treatment regimen, intravesical administration of rBCG-IFN-*γ* significantly prolonged animal survival compared to medium-treated controls, whereas BCG carrying an empty vector only slightly increased survival. In a similar experiment using the MB49 syngeneic orthotopic tumor model in IFN-*γ* knockout mice, intravesical treatment with rBCG-IFN-*γ* failed to prolong survival of mice, indicating that rBCG-derived IFN-*γ* had no measurable antitumor effect in the absence of endogenous IFN-*γ*. Studies also provided the mechanisms underlying the effect of rBCG-IFN-*γ* on treating bladder cancer. As demonstrated, this rBCG-IFN-*γ* strain could specifically upregulate the expression of MHC class I molecules on MB49 cells *in vitro* compared to control BCG, as the MHC class I upregulation could be blocked by an inhibitory antibody to IFN-*γ*. This rBCG strain also enhanced recruitment of CD4^+^ T cells into the bladder and further induced the local expression of IL-2 and IL-4 mRNA compared to control BCG. In addition, we have also evaluated the effects of rBCG strains secreting mouse IL-2 or mouse IP-10 (a Th1 chemokine) on treating bladder cancer in the MB49 syngeneic orthotopic tumor model and observed survival benefits of these rBCG strains (our unpublished observations). All these observations suggest that rBCG strains secreting Th1 cytokines or chemokines possess improved antitumor properties and may offer new opportunities for the treatment of bladder cancer. 

Supporting Th1 cytokine-secreting rBCG, *Mycobacterium smegmatis* (*M. smegmatis*), a closely related nonpathogenic mycobacterial organism, has been engineered to secrete mouse TNF-*α* (*M. smegmatis*/TNF-*α*) and tested in a transplantable MB49 tumor model [128]. Studies demonstrated that lymphocytes from tumor-bearing mice vaccinated with *M. smegmatis*/TNF-*α* produced elevated and prolonged IFN-*γ* but no IL-10 in response to mycobacterial antigen or tumor lysate stimulation *in vitro*. Histopathology revealed significantly increased infiltrating CD3^+^ lymphocytes in the tumor nodules of mice receiving the recombinant vaccine compared to those of mice receiving wild-type bacteria. These observations indicated that *M. smegmatis*/TNF-*α* induced cell-mediated immunity. Importantly, mice implanted subcutaneously with MB49 tumor and treated at an adjacent site with the recombinant vaccine exhibited significantly reduced tumor growth with a 70% durable tumor-free survival compared to those treated with wild-type bacteria or BCG (a 10–20% long-term survival). Interestingly, treatment with *M. smegmatis*/TNF-*α* also resulted in similar tumor growth inhibition in T-cell-deficient athymic nude mice and reduced but not abolished tumor growth inhibition in NK cell-deficient Beige mice. These observations indicated that NK-cells contribute to the antitumor effect of *M. smegmatis*/TNF-*α* but are not solely responsible for the eradication of tumor. Like immunocompetent mice, Beige mice also developed tumor-specific memory after treatment with *M. smegmatis*/TNF-*α*. A study also demonstrated enhanced immunotherapeutic potential of a human TNF-*α* secreting recombinant *M. smegmatis* for treating bladder cancer [129]. The ability to deliver immunomodulatory cytokines with no pathogenic effects makes *M. smegmatis* attractive as an alternative intravesical mycobacterial agent for bladder cancer treatment.

## 6. Conclusion and Future View

Intravesical administration of live BCG for superficial bladder cancer is the most successful immunotherapy for solid malignancy. However, BCG therapy is associated with significant toxicity and is ineffective in approximately 30–40% of cases. During the past 2 decades, advances in mycobacterial genetics and molecular biology have offered unprecedented opportunities for the development of genetically modified BCG strains that possess improved safety profile, immunogenicity, and protective efficacy. Among these, manipulation of BCG to secrete Th1 cytokines (e.g., IL-2, IL-18, IFN-*γ*, and IFN-*α*), alone or in combination with coexpression of bacterial or tumor antigens, represents one of the most attractive strategies for the development of improved vaccines. These types of rBCG strains have shown their potential to induce enhanced cellular immunity, leading to effective protection against mycobacterial infection (e.g., *M.tb*) and tumor progression (e.g., breast cancer) in various animal models. In bladder cancer treatment, BCG is administered intravesically; therefore, rBCG strains secreting Th1 cytokines can augment a localized cellular immune response that is crucial for effective BCG immunotherapy of bladder cancer. Since intravesical BCG in combination with local administration of Th1 cytokines such as rIFN-*α* has already been used in humans and demonstrated to be beneficial for bladder cancer patients, Th1 cytokine-secreting rBCG strains could be very useful as improved BCG agents. Indeed, these rBCG strains have been demonstrated to be capable of inducing antibladder cancer immune responses both *in vitro* and *in vivo* in animal studies. Because of their enhanced immunogenicity, Th1 cytokine-secreting rBCG strains can be used at a lower dose, potentially reducing side effects. Further studies should focus on determination of the clinically relevant effects of rBCG strains relative to each other and optimization of rBCG dosing and treatment schedule for each rBCG strain. Application of multiple rBCG strains should be tested and development of new rBCG strains continued. Moreover, the mechanisms underlying rBCG action need to be explored. Furthermore, influence of rBCG strains on Th17 and regulatory T (Treg) cells should be evaluated as the importance of these cell types in bladder cancer has being emerged. All these efforts will afford us a better understanding of Th1 cytokine-secreting rBCG strains and the steps necessary for use of the rBCG strains for treating bladder cancer. The pace of this research must be maintained if we are to improve this gold standard therapy for bladder cancer. Th1 cytokine-secreting rBCG strains merit further appraisal as improved BCG immunotherapeutic agents for the treatment of bladder cancer. 

## Figures and Tables

**Table 1:** Cytokine- and chemokine-expressing rBCG strains.

Strain	Cytokine	Species	Immunological effect	Reference
IL-2 BCG (RBD)	IL-2	m	Th1 cyt prod, Antitumor, Cytotoxicity	[30, 100, 101]
IL-2 BCG (MAO)	IL-2	r	Th1 cyt prod	[100]
BCG-CI	IL-2	h	Anti-BCG	[102]
BCG-CII	IL-2	h	Anti-BCG	[102]
BCG-IL-2	IL-2	m	CI, Th1 & Th2 cyt prod	[103]
BCG-GM-CSF	GM-CSF	m	CI, Th1 & Th2 cyt prod, DC act, Anti-*M.tb *	[103, 104]
BCG-IFN-*γ*	IFN-*γ*	m	CI, Th1 & Th2 cyt prod, Anti-BCG	[103, 105]
rBCG/IL-2	IL-2	m	CI, Th1 cyt prod, Anti-BCG	[106–108]
rBCG-IL-2/GFP	IL-2	m	CI, Th1 cyt prod, Anti-BCG	[109]
rBCG (*α*-Ag-IL-2)	IL-2	m	Th1 cyt prod, Cytotoxicity	[28]
BCG-IFN-*γ*	IFN-*γ*	m	Th1 cyt prod, Anti-BCG	[110]
rBCG-IFN-*α*	IFN-*α* 2B	h	Th1 cyt prod, Cytotoxicity	[38, 111, 112]
rBCG/IL-18	IL-18	m	no clear effect	[108]
BCG IL-18	IL-18	m	Th1 & Th2 cyt prod	[113, 114]
BCG-hIL2MUC1	IL-2	h	CI, Th1 cyt prod, Antitumor	[95, 96]
rBCG-IFN-*γ*	IFN-*γ*	m	CI, Th1 cyt prod, Antitumor	[115]
rBCG-IL-18	IL-18	m	Th1 cyt prod, Anti-BCG, Cytotoxicity	[29, 30]
rBCG-huIL-2-ESAT6	IL-2	h	CI, Th1 cyt prod, Cytotoxicity, HI	[116]
rBCG-IL-2	IL-2	h	Th1 cyt prod	[112]
BCGMCP-3	MCP-3	m	CI, Anti-BCG	[117]
rBCG-AEI	IFN-*γ*	m	CI, HI, Anti-*M.tb *	[118]
rBCG-Ag85B-IL15	IL-15	m	CI, Th1 cyt prod, Anti-*M.tb *	[119]
rBCG-MVNTR4-CSF	GM-CSF	h	CI, Th1 cyt prod, Antitumor	[97, 99]
rBCG-MVNTR8-CSF	GM-CSF	h	CI, Th1 cyt prod, Antitumor	[97, 99]
rBCG-Ag85B-Esat6-TNF-*α*	TNF-*α*	m	CI, HI	[120]

Anti-BCG: anti-BCG infection; Anti-*M.tb*: anti-*Mycobacterium tuberculosis *infection; CI: cellular immunity; DC act: dendritic cell activation; h: human; HI: humoral immunity; m: mouse; r: rat; Th1 cyt prod: T helper type 1 cytokine production; Th2 cyt prod: T helper type 2 cytokine production.
